# Persistent airway obstruction in severe eosinophilic asthma: targeting interleukin-5 and eosinophils

**DOI:** 10.1183/16000617.0024-2025

**Published:** 2025-12-10

**Authors:** Nicola A. Hanania, Felix J.F. Herth

**Affiliations:** 1 Section of Pulmonary, Critical Care and Sleep Medicine, Airways Clinical Research Center, Baylor College of Medicine, Houston, TX, USA; 2 Dept of Pneumology and Critical Care Medicine, Thoraxklinik and Translational Research Center, University of Heidelberg, Heidelberg, Germany

## Abstract

A large proportion of patients (40–60%) with severe asthma present with persistent airway obstruction (PAO). Patients with severe eosinophilic asthma (SEA) and PAO appear to be an underrepresented group in clinical guidance, despite being associated with considerable healthcare and economic burden. High levels of eosinophils drive airway inflammation, obstruction and hyperresponsiveness, which are key characteristics of SEA and can increase the risk of PAO and subsequent irreversible worsening of lung function. Available clinical and real-world data must examine the effectiveness of biologic treatment targeting SEA in PAO to identify any data gaps that might help further define such patients and optimise their management. However, clinical trials of SEA frequently exclude the enrolment of patients with PAO. Eosinophils are activated and recruited to airways in response to different cytokines, particularly interleukin-5 (IL-5), produced *via* a complex molecular and cellular cascade. A few studies evaluating the effectiveness of benralizumab and mepolizumab were able to identify cohorts of patients with SEA and PAO who had a significant response to these IL-5/Rα-targeted biologics. PAO in patients with SEA represents a distinct clinical entity, one which could be referred to as “persistent eosinophilic airway obstruction”. Patients with persistent eosinophilic airway obstruction are likely to be responsive to targeted biologic treatment, although additional clinical studies and real-world data are needed to further assess treatment efficacy and safety in this population and provide guidance to clinical practice.

## Shareable abstract

**Persistent airway obstruction in patients with severe eosinophilic asthma is a distinct clinical entity often referred to as “persistent eosinophilic airway obstruction” and is likely to be responsive to targeted biologic treatment.** https://bit.ly/4mpzc9I

## Introduction

Asthma is a chronic, inflammatory disease of the airways, currently estimated to affect 300 million people globally [1]. Asthma is typically characterised by airway hyperresponsiveness, chronic airway inflammation and reversible airway obstruction, which can become persistent [1]. Severe asthma, estimated to occur in approximately 4–8% of patients, is often uncontrolled despite optimal use of inhaled corticosteroids (ICS) and bronchodilators [2, 3]. Though asthma is often described as a heterogenous disease because of the diversity of possible underlying disease mechanisms, the majority of severe asthma cases are driven by eosinophilic airway inflammation (also known as type 2 high asthma) [1, 2, 4].

Severe eosinophilic asthma (SEA) is associated with increased risk of exacerbation, which in turn contributes to considerable healthcare resource utilisation and economic burden [2, 5, 6]. Indeed, stratifying patients according to blood eosinophil levels has shown that compared to those with normal eosinophil levels, patients with elevated blood eosinophil counts (≥150 cells·µL^−1^, ≥300 cells·µL^−1^ or ≥400 cells·µL^−1^) experienced significantly greater number of hospitalisations and incurred higher hospital costs [7–9]. In fact, healthcare resource use and costs associated with SEA are up to four times greater than for those without SEA [6].

These differences in healthcare utilisation and costs are largely driven by increased numbers of exacerbations [5, 9]. An analysis of 605 614 patients with asthma from the IBM MarketScan Research Databases determined that annual healthcare costs were around 1.5 times greater (USD 4384) for those with severe compared with nonsevere asthma, with asthma-related costs contributing approximately half of the difference. In those with severe asthma and exacerbations, costs were USD 5019 greater than those without exacerbations. Furthermore, patients with exacerbations were also more likely to have at least one inpatient admission or emergency department visit and greater average utilisation of outpatient services [9].

Asthma is associated with declining lung function over time [10]. Thus, while most patients with SEA meet the conventional definitions, some exhibit or go on to develop persistent airway obstruction (PAO). It is notable that SEA with PAO is a distinct clinical entity from airway diseases that exhibit features of both asthma and COPD (often referred to as asthma-COPD overlap or ACO) [11], but the differential diagnosis between these two clinical entities may be difficult to establish in clinical practice, particularly in patients who are older, smokers or have other risk factors for COPD [1]. Differential diagnosis may be further complicated by observations that eosinophilic inflammation may be present in 20–40% of patients with COPD and without a clinical history of asthma [12, 13]. Patients with eosinophilic COPD appear to be distinct from those patients with a history of asthma that develop COPD [13] and the phenotype of eosinophils appears to differ between eosinophilic COPD and asthma [14]. It has been suggested that certain features may help differentiate asthma with fixed airflow obstruction and COPD. These include levels of eosinophils or neutrophils in sputum and bronchiolar lavage, sputum cytokine profiles, transfer factor for carbon monoxide, fractional exhaled nitric oxide, high-resolution computed tomography (CT) emphysema score, reversibility to bronchodilators and steroids, the correlation between baseline reversibility to bronchodilators and forced expiratory volume in 1 s (FEV_1_) decrease rate, patient history, and the presence of comorbidities or risk factors [1, 11, 15]. There has been much discussion around ACO, in particular the lack of a universally accepted definition and the heterogeneity of individuals included, which has been reviewed in detail elsewhere [16–18]. Similarly, the concept of eosinophilic COPD has also been discussed in detail elsewhere [19].

A cohort study of adult patients with SEA identified that the presence of PAO was a significant independent predictor of uncontrolled asthma and nonresponse to biologic treatment [20, 21]. While current asthma guidelines including the Global Initiative for Asthma strategy offer guidance on patient characteristics that predict optimal response to anti-interleukin (IL)-5 and anti-IL-5 receptor α (anti-IL-5Rα) biologics, they do not provide any information specifically addressing patients with SEA and PAO [1].

## Aims

- Highlight the need for further classifying patients with severe asthma according to markers and clinical criteria to ensure tailored treatment strategies, introducing the concept of patients with SEA and PAO.
- Review the clinical characteristics and risk factors associated with the development of PAO in patients with asthma.
- Understand the disease mechanisms involved in the airway inflammation characteristic of severe asthma and particularly the role of eosinophils.
- Explore the clinical trial data and real-world evidence of IL-5 targeted biologic therapies in this patient population, highlighting the need for further studies.

## Methods

This narrative review was informed by literature searches conducted in the PubMed database. Searches, restricted to English language only, and with a focus on articles published in the past 10 years, were conducted using variants of the following terms and various combinations thereof: “asthma COPD overlap”, “asthma COPD overlap syndrome”, “ACOS”, “ACO”, “persistent airflow limitation”, “persistent airway limitation”, “persistent airflow obstruction”, “persistent airway obstruction”, “fixed airflow obstruction”, “fixed airway obstruction”, “severe asthma” and “severe eosinophilic asthma”.

Randomised controlled trials and subsequent analyses, observational studies, translational studies, and systematic and narrative reviews were retrieved. Titles and abstracts of articles arising from various search strings underwent an initial screening to identify suitable references which were then scrutinised further from a full-text review to determine their relevance for inclusion in the different sections and topics covered in this narrative review article. Additional publications were identified from the reference lists of retrieved articles. Review articles on this topic were also identified from the literature searches and screened to identify additional publications that had been cited. The use of the various search terms and decision to include papers from the past 10 years was made to ensure a comprehensive analysis of the literature, while recognising the lack of formal definitions and varying terminology in the literature.

## Prevalence, clinical characteristics and risk factors

Patients with PAO in SEA have diminished lung function, with post-bronchodilator ratio of FEV_1_ to forced vital capacity (FVC) lower than normal limits (*i.e.* ≤0.7) and often have poorer bronchodilator reversibility than is typically observed in other patients with asthma [2, 22]. It is important to recognise that bronchodilator reversibility may change from day to day in the same patient and may differ with different testing methods or following biologic treatment [1, 2]. There is no clear consensus definition for this patient group. Although some studies define PAO as a post-bronchodilator FEV_1_/FVC ≤0.7 [2, 22, 23], others state higher values [24], do not state the cut off for the lower limit of normal [25, 26], or require multiple consecutive occurrences [27]. Many studies have also included other parameters in their definition, such as an FEV_1_ <70% or 80% pred [28–30] or a total lung capacity >75% pred [31]. Most studies also required the specified lung function parameters to be observed on multiple occasions in order to meet the definition of PAO [24, 27, 29, 30]. Nevertheless, persistence of airflow obstruction and the presence of chronic eosinophilic inflammation should be considered hallmarks of PAO in SEA. As exposure to noxious particles is often considered with the diagnosis of COPD, when smoking history is taken into account, these patients may often be misdiagnosed with COPD or ACO [32].

A lack of consistent definition for PAO between studies may confound an accurate understanding of its prevalence in asthma patients and as such estimates may vary. The prevalence of PAO in the asthma population varies but is greater in those with severe disease. In nonsevere asthma, studies report that 16–24% of the patient population had PAO, whereas in severe asthma, as many as 40–60% had PAO [21, 27, 28, 31, 33–35]. PAO leads to worse clinical outcomes in patients with asthma, including increased exacerbation frequency, hospitalisation and emergency room visits, decreased lung function, and greater mortality [22, 25, 36].

A number of studies over the years have identified a range of risk factors associated with the development of PAO in asthma (table 1); however, these studies differ in cohort sizes, methodology and definitions of PAO used, and some parameters identified have been found to increase risk in some studies, or confer no increased risk in other studies [26, 35]. Risk factors identified according to clinical characteristics and demographics have included poor baseline lung function [28, 30, 34], small airways dysfunction [37], longer disease duration [27–29, 33, 35, 37–40], more exacerbations [37], male sex [27, 28, 35, 37, 38], older age [27, 37–39, 41] and history of smoking [27, 28, 35, 40]. Risk factors related to key biomarkers of inflammation have included high concentrations of eosinophils in sputum and/or blood [28, 30, 31, 35, 37], sputum periostin [24], serum eosinophil cationic protein and urinary eosinophil-derived neurotoxin [26], and increased neutrophils in sputum and/or blood [28, 30]. Airway remodelling may develop as a response to chronic eosinophilic inflammation, and this may contribute to airflow obstruction in SEA. Indeed, risk factors associated with remodelling include bronchial wall thickening (airway smooth muscle and reticular basement membrane) [29]. Higher levels of certain sputum biomarkers (fibroblast growth factor-2 and granulocyte-macrophage colony-stimulating factor) have been shown to be inversely correlated with lung function parameters and airway obstruction [1, 24]. Conversely, a decreased risk of PAO is associated with rhinitis, atopy, atopic dermatitis, higher education and Hispanic ethnicity [27, 35].

**TABLE 1:** Risk factors for persistent airway obstruction (PAO) according to clinical characteristics and demographics and biomarkers, which have been positively identified in various studies since 2001

Clinical characteristics and demographics [27–31, 33, 34, 37–41]	Biomarkers [24, 26, 28–31, 37, 39, 40]
**Clinical characteristics**	**Inflammation**
Poor lung function	Higher sputum concentrations of:
Lower FEV_1_% pred	Periostin
Lower bronchodilator responsiveness	TGF-β
Less reversibility	Eosinophils
Longer disease duration	Neutrophils
Worse asthma control	Higher serum concentrations of:
Higher exacerbation rate	Eosinophils
More hospitalisations	Neutrophils
Absence of rhinitis	Periostin
Absence of atopy	Increased urinary EDN
Atopic dermatitis	Higher *F*_ENO_
Aspirin sensitivity	**Remodelling**
**Demographics**	Bronchial wall thickening
Male gender	Increased airway smooth muscle area
Older age/adult onset	Reticular basement membrane thickening
Lower BMI	
African-American ethnicity	
Smoking history (past exposure; current use; past use)	
Exposure to domestic, visible mould	

BMI: body mass index; EDN: eosinophil-derived neurotoxin; *F*_ENO_: fractional exhaled nitric oxide; FEV_1_: forced expiratory volume in 1 s; TGF: transforming growth factor.

## Pathophysiology – role of eosinophils in severe asthma

PAO in asthma develops over many years, resulting from chronic airway inflammation and the subsequent continued remodelling that leads to thickening of the airway walls and parenchymal lung damage, and ultimately loss of elastic recoil [42]. The airway inflammation characteristic of severe asthma is determined by the types of inflammation present. Type-2 inflammation (type 2-high) is typically characterised by elevated blood eosinophil counts and/or increased fractional exhaled nitric oxide (*F*_ENO_) [43, 44]. The risk of developing PAO is greater for patients with eosinophilic asthma than for asthma patients with low eosinophil counts, suggesting an important role for this cell in disease pathophysiology [45]. Indeed, high variability in sputum eosinophils and increased eosinophils in blood have been linked to progressive declines in lung function [46–48]. Interestingly, the addition of tiotropium to ICS–long-acting β_2_-agonist (LABA) in patients with asthma has been reported to improve airflow obstruction, an effect that was correlated with reduced airway thickness and occurred without changes in *F*_ENO_ [49], suggesting that inflammation may not be the only driver of airway remodelling and PAO.

Eosinophils are multifunctional leukocytes that are found in many locations throughout the healthy body and play an important role in homeostasis (such as fat deposition, glucose homeostasis and tissue regeneration), as well as immunity (defence against pathogens and parasites, antigen presentation, and thymic T-cell selection) [50–52]. However, eosinophils can undergo a maladaptive response called “eosinophilic immune dysfunction” and become pathogenic in diseases such as severe asthma, contributing to disease pathogenesis through the release of toxic mediators; furthermore, the release of pro-inflammatory cytokines leads to the increased recruitment, infiltration and survival of eosinophils [50, 53].

**FIGURE 1:**
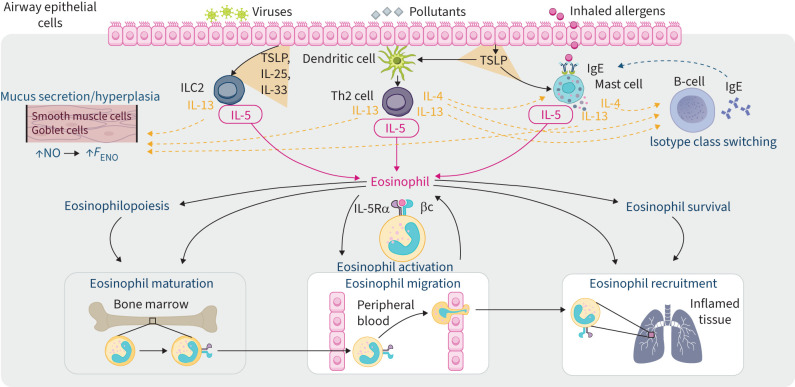
Eosinophil recruitment and activation in severe eosinophilic asthma and the role of IL-5. Adapted with permission from [44]. *F*_ENO_: fractional exhaled nitric oxide; IgE: immunoglobulin E; IL: interleukin; IL-5Rα: interleukin-5 receptor alpha; ILC2: type 2 innate lymphoid cell; NO: nitric oxide; Th2: T-helper 2 cell; TSLP: thymic stromal lymphopoietin.

Eosinophil maturation, differentiation, activation, survival and recruitment to airways is largely directed by the inflammatory cytokine IL-5 [50–54], produced *via* a complex cellular and molecular cascade that is initiated when airway epithelial cells are triggered by exposure to inhaled allergens, pollutants or viral or bacterial pathogens (figure 1) [43, 44]. Epithelial damage, which may occur in response to exposure to allergens, results in the release of “alarmins” such as IL-25, IL-33 and thymic stromal lymphopoietin (TSLP), which trigger type 2 immunity through the activation and/or recruitment of a variety of inflammatory cells (reviewed by [55–57]). Activated airway epithelial cells release TSLP, which exerts effects on other cells within the pathway, such as type 2 innate lymphoid cells (ILC2), dendritic cells (which drive T-helper 2 (Th2) cells) and mast cells; IL-25 (also known as IL-17E) and IL-33 are also released, which additionally stimulates ILC2 cells [43, 44]. The release of alarmins can sequentially cause these cells to produce large quantities of IL-5 and IL-13 from ILC2 cells, IL-4, IL-5 and IL-13 from Th2 cells, and IL-4 and IL-5 from mast cells [43, 44, 54]. In addition, TSLP may act on airway smooth muscle cells to promote the expression of proinflammatory cytokines, such as IL-6, and chemokines, such as eotaxin [55, 58]. Alarmins such as IL-25 also stimulate smooth muscle cells to increase expression of extracellular matrix components [59], suggesting a potential direct role in airway remodelling [55]. Moreover, mast cells, basophils and eosinophils may also act as a source of further alarmin release (reviewed in [55–57]).

The cytokine IL-33 as well as TSLP are responsible for some of the typical pathophysiological effects of asthma [43]. IL-4/IL-13 act upstream in the cascade where they stimulate B-cells to undergo isotype class switching to produce IgE which itself sensitises mast cells to further allergen exposure and production of cytokines [43]. IL-4/IL-13 also impact lung airway structure through thickening of basal membrane, loss of epithelial structure and propagation of M2 macrophages, which can cause fibrosis [54]. IL-13 has multiple pro-inflammatory effects on airway remodelling including mucus hypersecretion and hyperplasia of smooth muscle and goblet cells in the airway, and enhances inducible nitric oxide-synthase activity, leading to increased levels of nitric oxide and, therefore, *F*_ENO_ [43, 54]. IL-4 is involved in activating inflammation in type 2-high asthma and stimulates mast cells to further release IL-4, IL-5 and IL-13 [43, 54]. IL-5 binds to the cell surface receptors on eosinophils, stimulating pathways leading to the transcription of genes involved in cell proliferation and survival [44]. Additional pleiotropic effects of IL-5 on eosinophils include promoting their maturation, activation, migration from bloodstream and recruitment to airways (figure 1) [44, 60].

After recruitment to inflamed tissues, eosinophils can lead to epithelial damage by oxidative stress, extracellular matrix disruption and cell cytotoxicity [53, 60]. In addition, eosinophils promote airway narrowing, hyperresponsiveness, fibrosis and long-term airway tissue remodelling, leading to loss of functional tissue [53, 60, 61]. Extracellular traps, Charcot–Leyden crystals and eosinophil peroxidase-generated oxidants cause airway eosinophils to form mucus plugs, further contributing to poor lung function and persistent airflow obstruction [43]. Mucus plugging is common in patients with severe asthma and can persist in the same lung segments for years [62, 63]. The link between mucus plugs and airflow obstruction in severe asthma was explored as part of a study in 146 patients with asthma and 22 control subjects; using a bronchopulmonary segment-based scoring system to assess mucus plugs on multidetector CT lung scans, it was proposed that lung segments are susceptible to mucus plugging due to heightened type 2 inflammation, as measured by sputum eosinophils and type 2 cytokines, and eosinophil-derived oxidants inducing stiffening of mucus gels [62]. Indeed, subsequent analyses have confirmed that mucus plug scores are negatively correlated with changes in FEV_1_ and FEV_1_/FVC and positively correlated with changes in blood and sputum eosinophils [63]. Elevated eosinophils in inflamed tissues and subsequent tissue remodelling and presence of mucus plugs contribute to the increased risk of PAO [42, 61]. Moreover, in addition to being associated with decreased lung function, the presence of mucus plugs in patients with asthma is associated with more frequent and more severe exacerbations [64].

## Treatment of PAO in SEA – the role of IL-5/Rα-targeted therapy

Biomarkers are key in the treatable traits concept for airway diseases, a multi-dimensional strategy that aims to achieve therapeutic precision by utilising individual phenotypic and endotypic features instead of assigning traditional, rigid, disease labels [18, 65]. The multifaceted effect of eosinophils in various diseases and, particularly, its central role in the eosinophilic phenotype of severe asthma, necessitates eosinophil depletion as a key component of the treatment strategy. The pivotal role of IL-5 and its downstream cascade in the pathophysiology of type 2-high asthma makes this cytokine and its receptor potential therapeutic targets to control severe and difficult-to-treat PAO in SEA.

There are currently three monoclonal antibodies available in clinical practice that target IL-5-related components of the eosinophilic pathway, namely mepolizumab and reslizumab (anti-IL-5), and benralizumab (anti-IL-5Rα) [66–71]. Mepolizumab is a humanised IgG1κ monoclonal antibody that contains specific murine antibody fragments directed against human IL-5 and reslizumab is a humanised IgG4κ monoclonal antibody that incorporates the complementarity-determining regions of JES1-39D10, a rat monoclonal IgG2a antibody that specifically binds to the human IL-5 epitope comprising amino acids 89–92. The specific and high-affinity binding to IL-5 prevents the cytokine from binding to its receptor, expressed on eosinophils, thereby inhibiting the initiation of cellular activity and maturation, proliferation and survival [43, 44]. Benralizumab is a humanised, afucosylated, monoclonal antibody that specifically binds to an epitope close to the binding site on IL-5Rα found primarily on eosinophils, basophils and on some other cell types, and consequently impedes IL-5 binding to this receptor on cells. Afucosylation of the Fc constant region of benralizumab also increases its affinity for the Fc receptor on natural killer cells, thereby recruiting natural killer cells to eosinophil (and basophil) targets that directly induce apoptosis *via* enhanced antibody-dependent cell mediated cytotoxicity. Consequently, there is a rapid and near complete depletion of eosinophils in the blood; sampling of airway tissue, sputum and bone marrow has confirmed near complete depletion in these relevant tissue compartments [72–77]. These three agents have been approved for the treatment of SEA based on a comprehensive clinical development programme (key clinical studies have been reviewed in detail elsewhere) [44, 78].

Despite the well documented efficacy and safety in the severe asthma patient population, there are limited trial data to inform clinical practice on the use of biologics in those patients with PAO, because such patients are largely excluded from randomised controlled trials. A cross-sectional analysis compared the baseline characteristics of patients in the Severe Heterogeneous Asthma Research collaboration Patient-centred (SHARP Central) registry with those of randomised controlled trials. It was shown that of the total of 1231 patients across 11 European countries in the registry, only 27% (n=327) were eligible for recruitment into randomised clinical trials [79], highlighting the difference between clinical trials and real-world clinical practice. Moreover, while several small studies and cohort analyses have described the findings from biologics in patients with asthma and features of COPD [80–84], further analyses of the databases used and study populations may be able to identify patients specifically with PAO and SEA to provide further insights into the benefits of biologic treatment.

Overall, therefore, there is further opportunity for data from real-world studies and registries to provide valuable information on patients with PAO in SEA. Analyses of clinical trials in SEA have identified subgroups of patients from the overall study population with characteristics consistent with PAO that can further inform clinical practice on the benefits of anti-IL-5/Rα directed therapy; however, these analyses, of which there are few, are largely confined to mepolizumab and benralizumab.

### Mepolizumab

A *post hoc* analysis of data from phase III studies (DREAM/MENSA [85, 86]) assessed the effect of mepolizumab in patients with SEA and features of PAO (FEV_1_/FVC <0.7; post-bronchodilator predicted FEV_1_ <80%, and poor bronchodilator reversibility (<12% or 200 mL)) (table 2) [87]. Compared with placebo, mepolizumab effectively reduced exacerbation rates in patients with SEA and PAO (DREAM: 3.05 with placebo *versus* 1.33 with mepolizumab; MENSA: 0.76 with placebo *versus* 0.35 with mepolizumab) and those with a smoking history (DREAM: 2.86 with placebo *versus* 1.29 with mepolizumab; MENSA: 2.02 with placebo *versus* 0.94 with mepolizumab) [87].

**TABLE 2:** Overview of studies with mepolizumab and benralizumab in patients with persistent airway obstruction (PAO) in severe eosinophilic asthma (SEA)

	Mepolizumab	Benralizumab	
	DREAM/MENSA [85–87]	SIROCCO and CALIMA [45]	MIRACLE^#^
**Study type**	*Post hoc* analysis of phase III randomised, placebo-controlled studies DREAM (NCT01000506) and MENSA (NCT01691521)	*Post hoc* analysis of pooled data from both phase III randomised, double-blind, parallel-group, placebo-controlled studies SIROCCO (NCT01928771) and CALIMA (NCT01914757)	*Post hoc* analysis of multicentre, randomised, double-blind, parallel group, placebo-controlled, phase III study MIRACLE (NCT03186209) in 79 centres in China (including mainland China and Taiwan), South Korea and the Philippines
**Patient population**	Patients with SEA and features consistent with fixed airway obstruction (≥40 years of age; FEV_1_/FVC <0.7, post-BD FEV_1_ <80%, poor BD reversibility of <12% or 200 mL)	Severe, uncontrolled asthma with baseline blood eosinophil counts of ≥300 cells·mL^−1^ (n=1493) PAO+: post-BD FEV_1_/FVC <70% (n=935) PAO−: post-BD FEV_1_/FVC ≥70% (n=558)	Patients aged 12–75 years with severe asthma, with baseline post-BD FEV_1_ lung function data and baseline blood EOS ≥150 cells·µL^−1^ and ≥300 cells·µL^−1^, according to PAO status PAO+: baseline post-BD FEV_1_/FVC <70% PAO−: baseline post-BD FEV_1_/FVC ≥70%
**Treatments**	DREAM: mepolizumab 750, 250, or 75 mg *i.v.* or placebo for 52 weeks MENSA: mepolizumab 75 mg *i.v.*, mepolizumab 100 mg *s.c.* or placebo for 32 weeks	Benralizumab 30 mg every 8 weeks or placebo for 48 or 56 weeks	Benralizumab 30 mg every 4 weeks for the first three doses and every 8 weeks thereafter or placebo over 48 weeks
**Outcomes assessed**	Exacerbation rates	Primary: annual rate of exacerbations Secondary: annual rate of exacerbations associated with ED visits or hospitalisations; changes from baseline at EOT for pre-BD and post-BD FEV_1_ (measured by spirometry), pre-BD and post-BD FVC, post-BD FEV_1_/FVC, TASS, ACQ-6 score, Asthma Quality of Life Questionnaire score, and percentages of patients who changed from PAO+ to PAO− or remained PAO+ at EOT	Primary: crude and annual asthma exacerbation rate Secondary: lung function (pre-BD FEV_1_) and TASS Additional secondary: ACQ-6 score, proportion of patients with ACQ-6 score ≥1.5 and SGRQ score

^#^: Kefang Lai, Guangzhou Institute of Respiratory Health, Guangzhou, China; personal communication. ACQ-6: six-item Asthma Control Questionnaire; BD: bronchodilator; ED: emergency department; EOS: eosinophils; EOT: end of treatment; FEV_1_: forced expiratory volume in 1 s; FVC: forced vital capacity; OCS: oral corticosteroid; PAO: persistent airway obstruction; SEA: severe eosinophilic asthma; SGRQ: St. George's Respiratory Questionnaire; TASS: total asthma symptom score.

Results from the multicentre MESILICO study in patients with late-onset SEA and PAO (n=47) have been published [23, 88, 89]. Criteria for patient recruitment to this study included SEA (blood eosinophils ≥150 cells·µL^−1^ at screening or ≥300 cells·µL^−1^ the last 12 months) and PAO (post-bronchodilator FEV_1_/FVC <0.7) [23]. In an analysis of 39 patients who completed 12 months of follow-up, bronchial biopsies showed that mepolizumab treatment resulted in improvements associated with airway remodelling [23], including significant reductions from baseline in basement membrane thickness, airway smooth muscle, epithelial damage extent and tissue eosinophil counts. These were accompanied by significant improvements in lung function parameters (FEV_1_, FVC) from baseline. Increased peak expiratory flow was positively correlated with reductions in airway smooth muscle layer thickness and epithelial damage [23, 88]. Further analysis of 45 patients completing 12 months of mepolizumab treatment showed a significant 67% reduction in severe disease exacerbation rate from baseline, accompanied by improvements in lung function and asthma control test score [23, 89]. These improvements were also shown to be maintained over 24 months [89]. As the inclusion criteria for this study included PAO, no comparisons with patients without features of PAO could be made in either of the analyses presented.

In a longitudinal analysis of patients with SEA in the Belgian Severe Astha Registry, anti-IL-5 treatment with mepolizumab during follow-up was associated with attenuated decline in FEV_1_ independently of variables such as exacerbations, smoking status or ICS dose [48]. Although the study could not confirm causality from this association, these data provide further support for the association between improvements in lung function and airway remodelling observed with mepolizumab in the MESILICO study [23]. Mucus plugging may also be a potential cause of PAO in SEA [42, 62] and a case study has suggested that mepolizumab may be effective in reducing mucus plugs [90]. However, data are currently limited and further studies are required to confirm an effect of mepolizumab on mucus plugs in patients with PAO in SEA.

### Benralizumab

**FIGURE 2:**
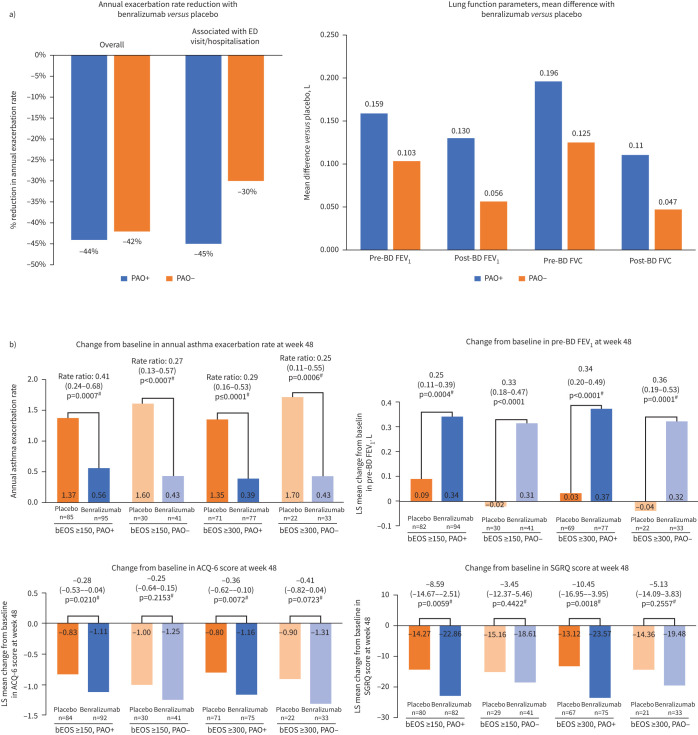
Clinical outcomes from studies with benralizumab in patients with severe eosinophilic asthma (SEA) with features typical of with persistent airway obstruction (PAO). a) SIROCCO/CALIMA study [45]. b) MIRACLE study. Data above columns in panel b) are rate ratio (95% confidence interval) or least squares mean difference between placebo and benralizumab (95% confidence interval). Adapted with permission from Kefang Lai, Guangzhou Institute of Respiratory Health, Guangzhou, China; personal communication. ACQ-6: Six-item Asthma Control Questionnaire; BD: bronchodilator; bEOS: blood eosinophils; ED: emergency department; FEV_1_: forced expiratory volume in 1 s; FVC: forced vital capacity; PAO: persistent airway obstruction; SEA: severe eosinophilic asthma; SGRQ: St. George's Respiratory Questionnaire. ^#^: Indicates nominal p-value not multiplicity protected.

The efficacy and safety of benralizumab were assessed in the SIROCCO and CALIMA phase III, international, multicentre, randomised, double-blind, parallel-group, placebo-controlled studies, in which patients aged 12–75 years with severe, uncontrolled asthma with eosinophilia were randomised (1:1:1) to receive benralizumab 30 mg every 4 weeks, 30 mg every 8 weeks or placebo for 48 weeks (SIROCCO) or 56 weeks (CALIMA) [72, 76]. A *post hoc* analysis of pooled data from these trials evaluated PAO status on treatment response in patients with baseline blood eosinophil counts of ≥300 cells·µL^−1^ who received benralizumab 30 mg every 8 weeks or placebo; PAO-positive or -negative status was defined as post-bronchodilator FEV_1_/FVC <70% and ≥70%, respectively (postbronchodilator reversibility was not accounted for in the definition of PAO status) (table 2) [45]. Using these criteria, PAO was present in almost two-thirds of the patient population (n=935/1493) with blood eosinophil counts of ≥300 cells·µL^−1^, evidencing an association between PAO and increased levels of eosinophils. Patients with PAO experienced improvements with benralizumab in asthma exacerbation rates (overall and associated with emergency department visits or hospitalisations), lung function, asthma symptoms and health-related quality of life *versus* placebo; these findings were comparable with those for patients without PAO (figure 2a) [45]. Of the patients with PAO at baseline, 21.6% of patients receiving benralizumab were no longer presenting with PAO at the end of treatment, compared with 15.4% with placebo [45]. These data specifically define a patient cohort with PAO in SEA that appears to be responsive to biologic therapy.

In a small (n=12) nonrandomised study of patients with SEA, reductions in sputum eosinophil levels, decreases in the number of mucus plugs and improvements in FEV_1_ have been observed following benralizumab treatment [91]. Similarly, another small nonrandomised study has confirmed a reduction in mucus plugs following benralizumab treatment [92]. These studies point to a potential mechanism of action for the benefits of benralizumab reported in the analysis of the CALIMA and SIROCCO, but these findings would need to be confirmed by further trials in patients with PAO in SEA.

The phase III placebo-controlled MIRACLE study assessed the efficacy and safety of benralizumab in an Asian population (n=695) with uncontrolled SEA despite receipt of medium-to high-dose ICS-LABA [93]. A *post hoc* analysis assessed effect of treatment in a subset of patients (n=453) with baseline post-bronchodilator FEV_1_ lung function data and baseline blood eosinophil counts ≥150 and ≥300 cells·μL^–1^ according to PAO-positive or -negative status (defined as post-bronchodilator FEV_1_/FVC <70% and ≥70%, respectively) (table 2) (Kefang Lai, Guangzhou Institute of Respiratory Health, Guangzhou, China; personal communication). In this Asian patient population, those who were PAO-positive had higher baseline blood eosinophils and worse lung function than those categorised as PAO-negative. At week 48, compared to baseline, benralizumab reduced annual asthma exacerbation rate and improved lung function, asthma control (assessed with the 6-item Asthma Control Questionnaire) and overall health status (assessed with the St George's Respiratory Questionnaire) irrespective of PAO status (figure 2b) (Kefang Lai, Guangzhou Institute of Respiratory Health, Guangzhou, China; personal communication). These data demonstrate the use of benralizumab in an Asian patient population and further support the efficacy of benralizumab in patients with PAO and SEA.

## Summary and future directions

Distinguishing cohorts of severe asthma patients with PAO from those without PAO but with other hallmarks of airflow obstruction remains challenging without a consensus on definitions and diagnostic criteria. We believe that this phenotype of severe asthma is clinically important and that a multifaceted and holistic diagnostic approach is encouraged to facilitate more accurate diagnoses, to enable optimal treatment pathways to be considered and drive improvement in treatment outcomes. Indeed, the benefits of anti-IL-5 treatment may vary between patients with SEA with PAO and those with other airway obstruction diseases such as eosinophilic COPD. Reductions in exacerbations following treatment with anti-IL-5 agents mepolizumab and benralizumab are small, with limited evidence on lung function improvements [94–96]. These findings contrast with the improvements observed with mepolizumab and benralizumab in patients in patients with SEA with PAO summarised herein, though further trials are clearly needed.

Patients with PAO in SEA represent a distinct clinical entity and, to facilitate this, a new terminology for this important group should be proposed, “persistent eosinophilic airway obstruction”. These patients experience increased disease severity (symptoms and exacerbations), more comorbidities, higher healthcare costs, poorer quality of life and higher mortality rates compared with those with asthma. SEA with PAO represents a progressive condition, with mucus plugs developing and stiffening, and airways undergoing remodelling leading to an increasing burden of symptoms and risk of exacerbations. Therefore, identifying and treating these patients early is important for minimising symptoms, improving quality of life and reducing the risk of exacerbations. However, the exclusion of these patients from the majority of clinical trials, combined with the limited number of specific interventional studies in this cohort, has resulted in a lack of knowledge of effective treatment strategies to guide clinical practice and provide the optimal outcomes. Identification of patients specifically with PAO and SEA in real-world studies and registries would provide informative data on this specific patient cohort going forwards. However, the lack of a clear consensus on the definition and diagnostic criteria hinders the inclusion and interpretation of clinical trials and observational studies to assess treatment options for these patients. There is therefore a clear need for experts and societies to provide recommendations and guidance on the diagnosis and definition of SEA with PAO so that they can be included or better represented in future studies assessing potential treatment options. In the interim, the pathophysiology of SEA indicates the role eosinophils and inflammatory cytokines, such as IL-5, play in contributing to airway and smooth muscle dysfunction, and available clinical and real-world analyses to date have shown that these patients with PAO respond to treatment, including with the biologics approved for SEA. Establishing PAO in SEA as a distinct clinical entity is important to advance the understanding and management of these patients.

**Points for clinical practice**

- A range of patients with severe asthma (40–60%) also present with PAO and are an underrepresented group in clinical guidance, but one which is associated with considerable healthcare and economic burden.
- It is important to recognise that the prevalence of PAO in the asthma population varies but is greater in patients with severe disease and a thorough assessment of clinical characteristics is necessary to ensure timely identification of such patients.
- PAO in patients with SEA represents a distinct clinical entity, one which could be referred to as “persistent eosinophilic airway obstruction”. Patients with persistent eosinophilic airway obstruction are likely to be responsive to targeted biologic treatment.
